# Research on Deflection and Stress Analyses and the Improvement of the Removal Uniformity of Silicon in a Single-Sided Polishing Machine Under Pressure

**DOI:** 10.3390/mi16020198

**Published:** 2025-02-08

**Authors:** Guoqing Ye, Zhenqiang Yao

**Affiliations:** School of Mechanical Engineering, Shanghai Jiao Tong University, Shanghai 200240, China

**Keywords:** chemical–mechanical polishing (CMP), small deflection, von Mises stress, pressure ring, uniformity

## Abstract

The chemical–mechanical polishing (CMP) of silicon wafers involves high-precision surface machining after double-sided lapping. Silicon wafers are subjected to chemical corrosion and mechanical removal under pressurized conditions. The multichip CMP process for 4~6-inch silicon wafers, such as those in MOSFETs (Metal Oxide Semiconductor Field Effect Transistors), IGBTs (Insulated-Gate Bipolar Transistors), and MEMS (Micro-Electromechanical System) field materials, is conducted to maintain multiple chips to improve efficiency and improve polish removal uniformity; that is, the detected TTV (total thickness variation) gradually increases from 10 μm to less than 3 μm. In this work, first, a mathematical model for calculating the small deflection of silicon wafers under pressure is established, and the limit values under two boundary conditions of fixed support and simple support are calculated. Moreover, the removal uniformity of the silicon wafers is improved by improving the uniformity of the wax-coated adhesion state and adjusting the boundary conditions to reflect a fixed support state. Then, the stress distribution of the silicon wafers under pressure is simulated, and the calculation methods for measuring the TTV of the silicon wafers and the uniformity measurement index are described. Stress distribution is changed by changing the size of the pressure ring to achieve the purpose of removing uniformity. This study provides a reference for improving the removal uniformity of multichip silicon wafer chemical–mechanical polishing.

## 1. Introduction

The chemical–mechanical polishing of silicon wafers involves the combined action of chemical corrosion and mechanical removal under pressure [1,2,3]. A multihead chemical–mechanical single-sided polishing machine involves plate rotation, and four polishing heads independently drive rotation in the same direction and apply pressure to the workpiece for polish removal [4,5].According to the classical Preston equation MRR = KPV [6,7] for grinding, the material removal rate (MRR) is proportional to the pressure P, so the uniformity of pressure distribution directly affects the uniformity of removal. For large silicon wafers, such as 12-inch silicon wafers, regional pressure control is commonly applied when the silicon wafer is single-side-polished by attaching a vacuum cup to the tool [8,9,10,11]; however, for small silicon wafers ranging from 4 to 6 inches [12], removal uniformity is improved by reducing small deflection deformation through the boundary conditions of wax-coated, bonded silicon wafers and making pressure distribution more uniform by changing the size of the pressure ring [13]. The multichip CMP process for small silicon wafers, such as those in MOSFETs (Metal Oxide Semiconductor Field Effect Transistors), IGBTs (Insulated-Gate Bipolar Transistors), and MEMS (Micro-Electromechanical System) field materials, is conducted to maintain multiple chips to improve efficiency and improve polish removal uniformity; that is, the detected TTV gradually increases from 10 μm to less than 3 μm. In this work, first, a mathematical model of the small deflection [14] analysis of the thin plate [15] of a silicon wafer is established, and the deflection under fixed and simple support boundary [16,17] conditions is calculated. The deflection value under actual engineering conditions is between the two ideal boundary conditions. Moreover, small deflection deformation is improved by the boundary conditions of the wax-coated, bonded silicon wafers, and removal uniformity is improved in these experiments. Then, a stress distribution simulation of the force model is performed, and a TTV calculation formula is established. On the basis of TTV data analysis, the judgment conditions of the pressure ring that need to be applied are obtained, and a calculation formula for the pressure ring change is developed. Moreover, the stress distributions of the two pressure ring conditions were compared and simulated; index λ of the pressure ring change formula was obtained, and it was verified that a change in the pressure ring could improve the stress distribution and uniformity of polish removal [18,19].

## 2. Methodology

### 2.1. Silicon Compression Structure Model

**Figure 1:**
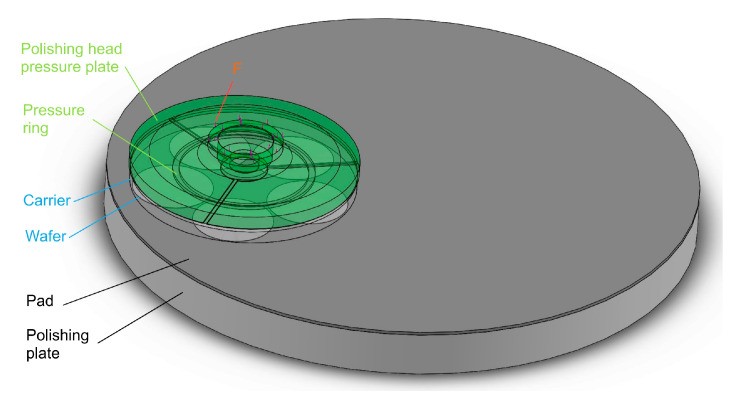
Silicon wafer compression structure model.

As shown in Figure 1, in the silicon wafer compression structure model of a small-sized (from 4 inches to 6 inches), silicon wafer, single-sided polishing machine, the bottom is composed of a polishing plate and a polyurethane polishing pad, and the polyurethane polishing pad is glued to the polishing plate on one side [20,21]. The middle part is composed of a SiC carrier and a workpiece silicon wafer, and the silicon wafer is attached to the carrier via automatic coating equipment [22]. The upper part is composed of a polishing head pressure plate and a pressure ring. The pressure ring is a polyurethane single-sided adhesive attached to the bottom of the polishing head pressure plate. The bottom, middle, and top are marked with three different colors. The silicon wafer attached to the SiC carrier was pressurized downwards with a uniform load F from the polishing head. Since the four polishing heads are centrally symmetrical, only one was analyzed.

### 2.2. Calculation of the Small Deflection Model of a Silicon Wafer

Since the polishing pad is a polyurethane elastic pad, which is regarded as an elastic half space, the silicon wafer is considered smooth after being lapped by a double-sided lapping machine [23,24] before chemical mechanical polishing and is fully in contact with the polyurethane polishing pad after loading. The silicon wafer material is monocrystalline silicon, which is anisotropic, and the elastic circular sheet here is regarded as isotropic [25], which has a slight influence on the calculation accuracy. The wafer thickness is t = 0.5, and the diameter is 125 mm. t/b < 1/5 for a thin plate [26,27]. The silicon wafer is prepared to adhere to the same carrier as a group of uniform thicknesses through thickness-sorting equipment and is coated with wax under the ceramic carrier of a high-precision plane by professional equipment. Therefore, a single silicon wafer is regarded as a uniform load. The bending method of a thin circular plate related to elasticity is used to analyze the small deflection of a silicon wafer under pressure. In addition, because the positioning edge of a silicon wafer has many forms, the model is simplified to a circle without a positioning edge for analysis.

**Figure 2:**
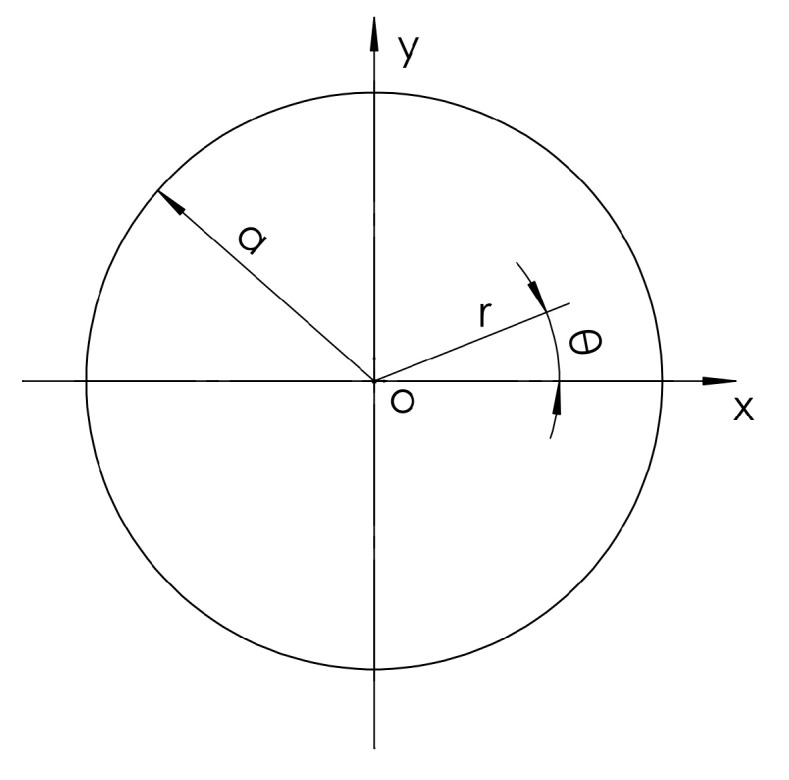
Coordinate chart of the elastic circular thin-plate model of a silicon wafer.

As shown in Figure 2, according to the basic equation of small deflection of rigid thin plates, the Sofi Germain equation is as follows:

(1) $$\nabla^{4}=\left(\frac{\partial^{4}}{\partial x^{4}}+2\frac{\partial^{4}}{\partial x^{2}\partial y^{2}}+\frac{\partial^{4}}{\partial y^{4}}\right)w=\frac{q}{D}$$

where $D=\frac{Et^{3}}{12(1-v^{2})}$.

In the formula, the Cartesian coordinate system is adopted, where *w* is the deflection, *q* is the load, *D* is the stiffness, *E* is the elastic modulus of the silicon wafer, and *v* is the Poisson’s ratio of the silicon wafer.

Using the transformation relationship between the Cartesian coordinate system and polar coordinates (*r*, *θ*), basic Equation (1) of the thin plate can be written as

(2) $$\nabla^{4}w={\left(\frac{\partial^{2}}{\partial x^{2}}+\frac{\partial^{2}}{\partial r^{2}}\right)}^{2}w={\left(\frac{\partial^{2}}{\partial r^{2}}+\frac{1}{r}\frac{\partial }{\partial r}+\frac{\partial^{2}}{r^{2}\partial \theta^{2}}\right)}^{2}w=\frac{q}{D}$$

The model considers the downwards pressure of the silicon wafer transmitted by pressure *F* to the carrier as uniformly distributed, so it is an axisymmetric load independent of *θ*. The supporting conditions of the silicon wafer are also axisymmetric, so the problem is independent of *θ*. Formula (2) can be further written as follows:

(3) $$\left(\frac{d^{2}}{dr^{2}}+\frac{1}{r}\frac{d}{dr}\right)\left(\frac{d^{2}w}{dr^{2}}+\frac{1}{r}\frac{dw}{dr}\right)=\frac{1}{r}\frac{\partial }{\partial r}\left\{r\frac{d}{dr}\left[\frac{1}{r}\frac{d}{dr}(r\frac{dw}{dr})\right]\right\}=\frac{q}{D}$$

Equation (4) can be obtained by integrating Equation (3):

(4) $$w=C_{1}+C_{2}lnr+C_{3}r^{2}+C_{4}r^{2}lnr+\frac{qr^{4}}{64D}$$

where *C*_1_, *C*_2_, *C*_3_, and *C*_4_ are arbitrary integral constants. Since the deflection of each point of the thin plate is limited, for the solid thin plate, the value of the deflection *w* cannot be infinite at the center of the plate, that is, where *r* = 0. The two coefficients *C*_2_ and *C*_4_ related to ln*r* in Equation (4) should be equal to 0; then, Equation (4) becomes the following:

(5) $$w=C_{1}+C_{3}r^{2}+\frac{qr^{4}}{64D}$$

According to the thin-plate boundary condition, when the deflection *w* on the boundary is zero and the normal angle is zero, it is the fixed support boundary.

(6) $${\left.w\right|}_{r=a}=0,\;{\left.\frac{\partial w}{\partial r}\right|}_{r=a}=0$$

Equation (6) is substituted into Equation (5) to obtain Equation (7).

(7) $$\left\{\begin{array}{c} C_{1}+C_{3}a^{2}+\frac{qa^{4}}{64D}=0\\ {2C}_{3}a+\frac{qa^{3}}{16D}=0 \end{array}\right.$$

By solving Equation (7), we obtain the following:

$$C_{1}=\frac{qa^{4}}{64D},\;C_{3}=-\frac{qa^{2}}{32D}$$

Substituting the values of *C*_1_ and *C*_3_ into (5) yields the following:

(8) $$w=\frac{qa^{4}}{64D}{\left(1-\frac{r^{2}}{a^{2}}\right)}^{2}$$

When *r* = 0, that is, at the center of the circle, *w* is the maximum value, which is as follows:

(9) $$w_{max}={\left.w\right|}_{r=0}=\frac{qa^{4}}{64D}$$

In the other case, according to the thin-plate boundary condition, the simply supported boundary is when the deflection *w* at the boundary is zero and the bending moment *M_N_* in the direction of normal *N* is zero.

$${\left.w\right|}_{r=a}=0,\;{\left.M\right|}_{r=a}={\left.-D(\frac{d^{2}w}{dr^{2}}+\frac{v}{r}\frac{dw}{dr})\right|}_{r=a}=0$$

By substituting the above equation into Equation (5), the following equations are obtained:

(10) $$\left\{\begin{array}{c} C_{1}+C_{3}a^{2}+\frac{qa^{4}}{64D}=0\\ {2(1+v)C}_{3}+(3+v)\frac{qa^{2}}{16D}=0 \end{array}\right.$$

By solving Equation (10), we obtain the following:

$$C_{1}=\frac{(5+v)qa^{4}}{64(1+v)D},\;C_{3}=-\frac{(3+v)qa^{2}}{32(1+v)D}$$

Plug them into (5) to obtain the value of *w*

(11) $$w=\frac{qa^{4}}{64D}(1-\frac{r^{2}}{a^{2}})(\frac{5+v}{1+v}-\frac{r^{2}}{a^{2}})$$

When *r* = 0, that is, at the center of the circle, *w* is the maximum value.

(12) $$w_{max}={\left.w\right|}_{r=0}=\frac{(5+v)qa^{4}}{64(1+v)D}$$

The actual boundary conditions of silicon wafers are not idealized fixed support and simply supported states.

The deflection at the center of the silicon wafer is the largest, and the deflection value is within the interval of two ideal states.

(13) $$\frac{qa^{4}}{64D}\leq w\leq \frac{(5+v)qa^{4}}{64(1+v)D}$$

where $\;D=\frac{Et^{3}}{12(1-v^{2})}$.

The parameters of the 5-inch silicon wafer are as follows:

E = 1.9×1011 Pa, v = 0.278, t = 0.5 mm, q = 16,305 Pa, and a = 62.5 mm.

By substituting these values into Equations (9) and (12), the *w* values under the two boundary conditions are *w*_1_ = 0.34 μm and *w*_2_ = 1.36 μm, respectively.

Therefore, the deflection range of the silicon wafer is

0.34 μm≤w≤1.36 μm

Equations (9) and (13) indicate that the elastic modulus E and Poisson’s ratio *v* are determined when the silicon wafer material is used, and the thickness t and radius a of the silicon wafer of a certain specification are also determined. Therefore, the deflection is proportional to the loaded load. Reducing the deflection can reduce the load q, but reducing the load affects the removal rate of the material. Therefore, a balance needs to be struck in terms of the material removal rate and deflection accuracy. For 5-inch silicon wafers, the difference in deflection between the two boundary conditions of fixed support and simple support is that the fixed support is 1/4 of the simple support, so a good wax attachment state is closer to the fixed support state, which can reduce the deflection and improve the removal uniformity.

**Figure 3:**
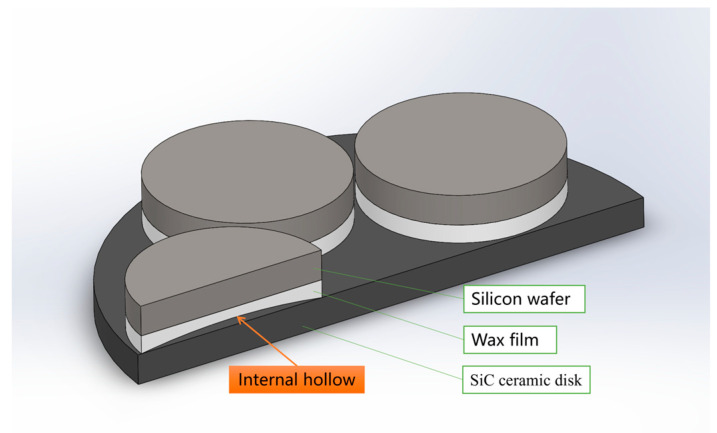
Diagram of waxing attached to a silicon wafer.

In Figure 3, five silicon wafers are attached to a SiC ceramic plate by a heated wax film. The wax film is coated on the silicon wafer and then turned and attached to the SiC ceramic substrate. In this process, a slightly convex shape will appear on all sides, and the internal hollow form will be formed after affixing. Therefore, to improve the wax-coated adhesion state, that is, to change the adhesion boundary conditions of the silicon wafer, it can be flatter and tend to follow the fixed support boundary conditions that, as per the calculation results, reduce the deflection. The closer the deflection is to the fixed support state of 0.34 μm, the more advantageous it is to control the polishing removal uniformity target within 3 μm. In this study, this goal was achieved by optimizing the rotation speed of the wax coating to make the thickness of the wax film smaller and more uniform without causing the circumference to be slightly greater than the middle. This is described in the Results and Discussion.

### 2.3. Static Simulation of a Silicon Wafer Under Pressure

A static simulation analysis was carried out on the silicon wafer compression model. The materials of each component and the corresponding Young’s modulus E and Poisson’s ratio v are shown in Table 1.

The model is gridded, and a uniform load of F = 1000 N is loaded.

**Figure 4:**
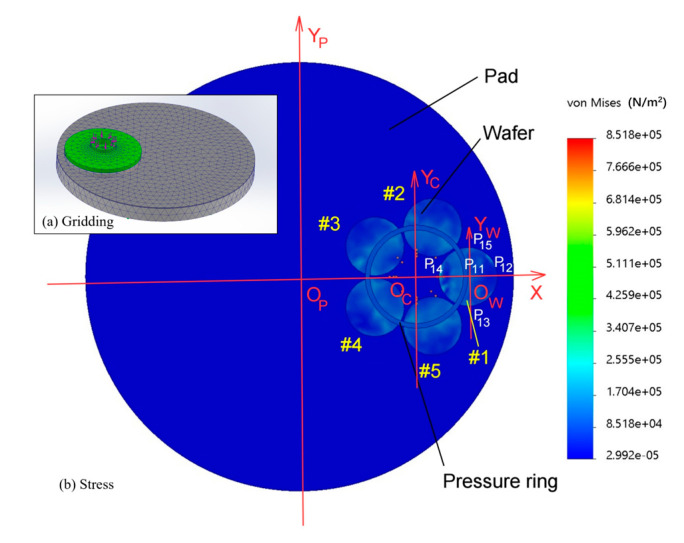
Von Mises stress.

As shown in Figure 4a, the main parameters of model meshing are as follows:

Grid type: solid grid;
Meshes used: meshes on the basis of mixed curvature;
Jacobi points for high-quality grids: 16 points;
Maximum unit size: 37.7542 mm;
Minimum unit size: 5.74025 mm;
Mesh quality: high;
Total number of sections: 28,778;
Total units: 15,867.

The resulting von Mises stress is displayed in Figure 4b.

Figure 4 shows the polishing pad, wafer, and pressure ring. An O_P_XY_P_ coordinate system is established with the center O_P_ of the polishing pad as the origin. The carrier is also associated with the pressure ring concentric Oc, which establishes the O_C_XY_C_ coordinate system for the origin. The OwXY coordinate system is established with the center Ow of the wafer as the origin.

The wafers are numbered #1~#5 counterclockwise. Five points are selected for each wafer and marked with wafer #1, as shown in Figure 4. Point 1 is the center O_W_ of the wafer, and point 2 is the far point away from O_C_. Point 4 is the closest point from O_C_; points 3 and 5 are equidistant from O_C_ and have angles of 270° and 90° from O_W_X, respectively; and points 2, 3, 4, and 5 are 6 mm from the edge of the wafer. The 1~5 points on the remaining #2–#5 silicon wafer are regarded as five circular arrays with O_C_ at the center of the circle on the #1 silicon wafer. Therefore, the center of all silicon wafers is point 1, point 2 is far from O_C_, point 4 is near O_C_, and points 3 and 5 are in the other direction of equal distance from O_C_. These points are numbered at the front point after the silicon wafer. The #1 wafers are numbered P11, P12, P13, P14, and P15. The #2 wafers are numbered P21, P22, P23, P24, and P25. There are a number of points on the #3, #4, and #5 wafers. These points are used as five-point measurement points for the silicon wafer TTV (total thickness variation) [28].

According to the von Mises detection data of the simulation results [29], the stress range was between 3 × 10^4^ Pa and 1.8 × 10^5^ Pa, and the stresses at points 1, 3, and 5 were essentially the same, whereas the stress at point 4 was greater than that at point 2. The stress distribution on a group of silicon wafers shows a centrosymmetric distribution with Oc at the center. This is mainly because the pressure ring pressurization method is adopted for the multichip polishing head, and the force state is centered on the pressure ring center O_C_ to pressurize several silicon wafers with symmetry in the center of this batch. To improve the TTV problem caused by nonuniform pressure, the measurement of the silicon wafer thickness and the calculation method of the TTV are discussed. On this basis, the calculation method and judgement model for adjusting the pressure ring are established.

### 2.4. Calculation Method and Analysis of the TTV of the Silicon Wafer Thickness

According to Figure 4, after the polishing head is pressurized by the pressure ring, the central symmetrical stress distribution with O_C_ as the center is formed on this group of silicon wafers, and the stresses at points 1, 3, and 5 are essentially the same; the stress at point 4 is greater than the stresses at points 1, 3, and 5; and the stress at point 2 is smaller than the stresses at points 1, 3, and 5. According to the Preston equation MRR = KPV, the different distributions of stress P affect the material removal rate (MRR) of silicon wafers, which causes differences in the thickness measured at five points on silicon wafers, and the difference between the maximum and minimum thicknesses measured is the TTV. To improve the TTV, that is, to reduce the difference, we need to adjust the stress to a more evenly distributed state. The following is a mathematical model of the TTV calculation and adjustment method to improve the TTV.

According to the wafer point position defined in Figure 4, the wafer thickness measured by the noncontact measuring instrument at wafer #1 points P_11_, P_12_, P_13_, P_14_, and P_15_ is set as t_11_, t_12_, t_13_, t_14_, and t_15_. Then, for wafer #2, the silicon wafer thickness measured by the noncontact measuring instrument at points P_21_, P_22_, P_23_, P_24_, and P_25_ is set to t_21_, t_22_, t_23_, t_24_, t_25_, etc. In this way, this group of five silicon wafers of the polishing head, with each silicon wafer in accordance with the five-point measurement method, is used to measure the thickness value. The following determinant is formed:

(14) $$\left[\begin{array}{ccc} t_{11} & \cdots & t_{15}\\ \vdots & \ddots & \vdots \\ t_{51} & \cdots & t_{55} \end{array}\right]$$

According to the definition of the TTV, the TTV value of silicon wafer #1 is denoted TTV_1_. TTV_1_ can be obtained by performing the following calculation on the first row of data of Equation (14).

(15) $${TTV}_{1}=Max\left(t_{11},t_{12},t_{13},t_{14},t_{15}\right)-Min\left(t_{11},t_{12},t_{13},t_{14},t_{15}\right)$$

Through each line of data, that is, the five-point thickness measured on the same silicon wafer, we can obtain the TTV values of silicon wafers #2~#5, which are TTV_2_, TTV_3_, TTV_4,_ and TTV_5,_ respectively.

The average value of the TTV is denoted as $\bar{TTV}$.

(16) $$\bar{TTV}=({TTV}_{1}+{TTV}_{2}+\cdots +{TTV}_{n})/n$$

where n is the number of silicon wafers measured (*n* = 5 for this measurement).

According to the mean $\bar{TTV}$ and each TTV, the standard variance of the TTV can be obtained.

(17) $$\sigma =\sqrt{\frac{{{(TTV}_{1}-\bar{TTV})}^{2}+{{(TTV}_{2}-\bar{TTV})}^{2}+\cdots +{{(TTV}_{n}-\bar{TTV})}^{2}}{n-1}}$$

When the variance σ in the TTV value is small enough to meet the process requirement CPK (process capability index) [30], the consistency of the TTV data meets the standard. However, when the mean value of the TTV exceeds the technical requirements, the column data of Equation (14) need to be calculated and analyzed. Let the average of the first column be $\bar{t_{p1}}$

(18) $$\bar{t_{p1}}=(t_{11}+t_{21}+\cdots +t_{n1})/n$$

where $\bar{t_{p1}}$ is the average value of central point 1 of the measured silicon wafer.

By analogy, the average values of columns 2, 3, 4, and 5 are obtained as follows: $\bar{{\;t}_{p2}}\;,\bar{t_{p3}}\;,\bar{t_{p4}},\;\mathrm{and}\;\bar{t_{p5}}\;.$

This is to obtain the average of the points in the same position of each silicon wafer.

### 2.5. Method for Adjusting the Size of the Pressure Rings to Improve the Uniformity of the Pressure Distribution

If the corresponding points of the polishing head show the following conditions for more than three experiments, the pressure ring can be adjusted to improve the TTV.

(1) When the values of $\bar{t_{p1}}$, $\bar{t_{p3}}$, and $\bar{t_{p5}}$ are close, and $\bar{t_{p4}}<\bar{t_{p1}}<\bar{t_{p2}}$.

This indicates that point No. 4 near the carrier has the smallest thickness, and point No. 2 far from the carrier has the greatest thickness, whereas points No. 1, 3, and 5 are close to and between points No. 4 and 2. If this phenomenon is stable for more than three cycles, the stress at No. 4 is large, and the removal amount is the largest, whereas the stress at No. 2 is the smallest, and the removal amount is the smallest.

(2) When the values of $\bar{t_{p1}}$, $\bar{t_{p3}}$ and $\bar{t_{p5}}$ are close, and $\bar{t_{p4}}>\bar{t_{p1}}>\bar{t_{p2}}$.

This indicates that the thickness of point No. 4 near the carrier is the greatest, and the thickness of point No. 2 far from the carrier is the smallest, whereas points No. 1, 3, and 5 are close to and between points 4 and 2. If this phenomenon is stable more than three times, the stress at point 4 is small, and the removal amount is minimal, whereas the stress at point 2 is maximal, and the removal amount is maximal.

In general, technicians use an experience test pressure ring to increase or reduce the diameter and then test the thickness of the silicon wafer after each adjustment to determine the pressure ring size with a better result relative to the TTV or adjust it to meet the production requirements and do not find a better pressure ring diameter match. Different polishing heads of different equipment or the same equipment affect the pressure distribution in addition to the diameter of the pressure ring. There are other factors, such as the plate shape of the polishing head pressing plate and the plate shape of the lower polishing plate superimposed, so if the TTV detection requirements are not met, adjusting the pressure ring according to the difference in thickness of the silicon wafer at different points during equipment debugging may be necessary. In this way, the stress distribution can be improved to improve the thickness uniformity; that is, the TTV level can be increased. This method of pressure ring adjustment depends on experience and multiple tests, which costs time and requires many silicon wafers to test. Therefore, this work aims to establish a geometric model and mathematical calculation model of the thickness difference, and the diameter of the pressure ring that needs to be adjusted can be calculated according to the existing thickness measurement value.

**Figure 5:**
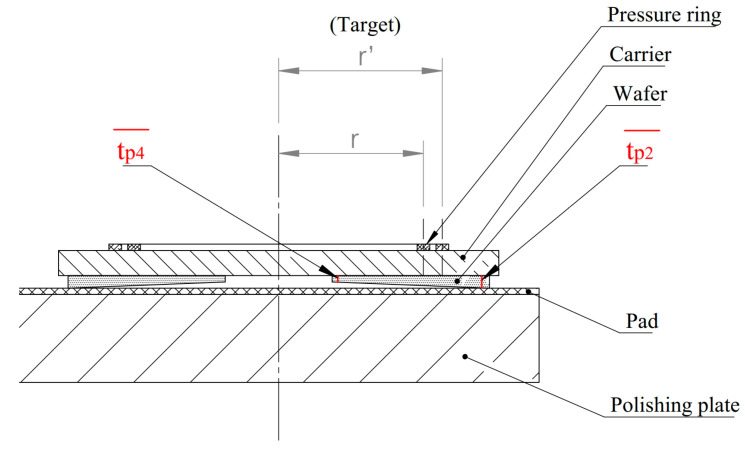
Pressure ring adjustment model diagram.

As shown in Figure 5, $\bar{t_{p4}}$ and $\bar{t_{p2}}$ are the average values of the center point of P_4_ near the center point of the pressure ring and P_2_ far from the center point of the pressure ring on the silicon wafer, respectively. Assuming that the silicon wafer is polished into a tilt, the value of $\bar{t_{p4}}$ in the figure is smaller than that of $\bar{t_{p2}}$; that is, the place near the center of the pressure ring is subjected to greater stress, resulting in greater removal of the polishing. The radius of the pressure ring in the current state is *r*, and the radius of the target pressure ring that needs to be adjusted is set to *r*’. The following mathematical model is constructed:

(19) $$\frac{r^{\prime }-r}{r}={\left(\frac{\bar{t_{p2}}-\bar{t_{p4}}}{\bar{t_{p4}}}\right)}^{\lambda }$$

where *r* and *r*’ are medium diameters, *λ* is the undetermined index, and the index can be adjusted according to different equipment environmental conditions. Solution (19) gives the following:

(20) $$r^{\prime }=\left[{\left(\frac{\bar{t_{p2}}-\bar{t_{p4}}}{\bar{t_{p4}}}\right)}^{\lambda }+1\right]r$$

## 3. Results and Discussions

To improve the TTV level of polished silicon wafers affected by pressure, the first step is to improve the uniformity of silicon wafer bonding so that the boundary conditions for deflection deformation after receiving pressure are close to those of fixed supports. If there is a tilt in a group of silicon wafers processed by individual polishing heads, a pressure ring adjustment method is used to further improve the removal uniformity.

**Figure 6:**
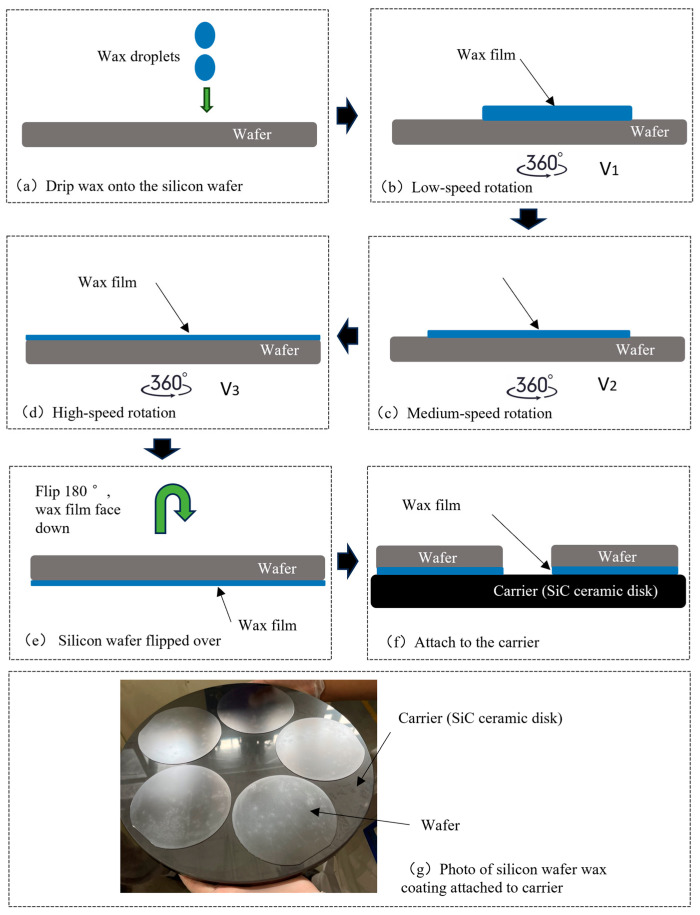
Process diagram for wax coating and attachment of silicon wafers to carriers.

As shown in Figure 6, the silicon wafer bonding process first involves dropping 1–2 mL of melted wax droplets onto the silicon wafer on the wax coating and attaching automation equipment (a). Then, by rotating the silicon wafer at low, medium, and high speeds (b–c–d three stages), the wax liquid is evenly dispersed under centrifugal force to form a wax film of approximately 1–2 μm. The silicon wafer is flipped 180° again, with the wax film facing downwards (e), and finally it is attached to the carrier (f). A photo of the silicon wafer after bonding is shown in (g).

**Figure 7:**
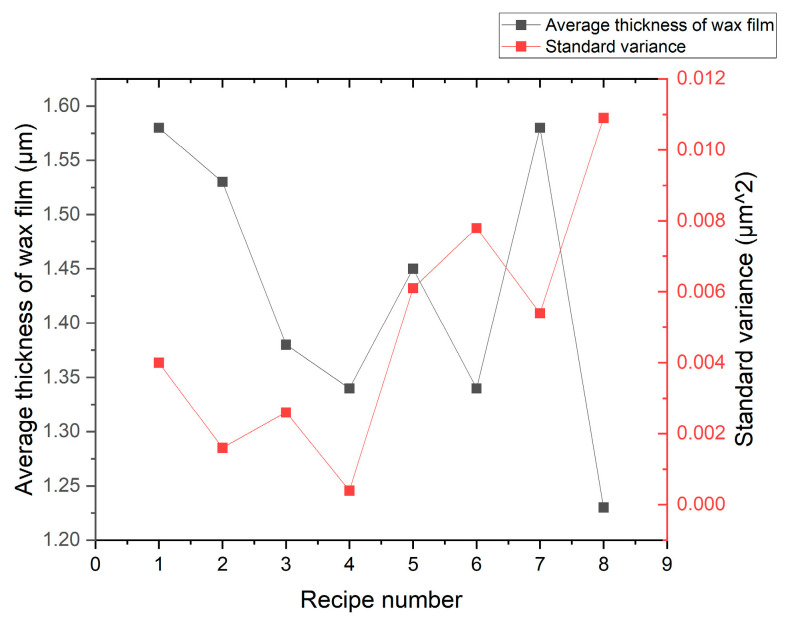
The average thickness and standard deviation of the wafer wax coating.

As shown in Figure 7, through the experimental comparison of eight process recipes for wax-coated silicon wafers, in recipe 4, that is, when the three-stage speed of the wax-coated coating is 1500 rpm, 2500 rpm, and 3000 rpm for 1, 2, and 3 s, respectively, the amount of wax drops per wafer is 1.5 mL, the optimal thickness and standard deviation of the wax layer are 1.3 μm, and the standard variance is only 0.0004 μm^2^. In this way, the silicon wafer is uniformly fitted with a ceramic disk, and boundary conditions approaching the fixed support are achieved; that is, under the processing force state, the silicon wafer is successfully fitted to the SiC ceramic disk. The deflection deformation also tends to be 0.34 μm under the fixed support boundary condition, which is conducive to achieving a removal uniformity deviation of 3 μm.

**Figure 8:**
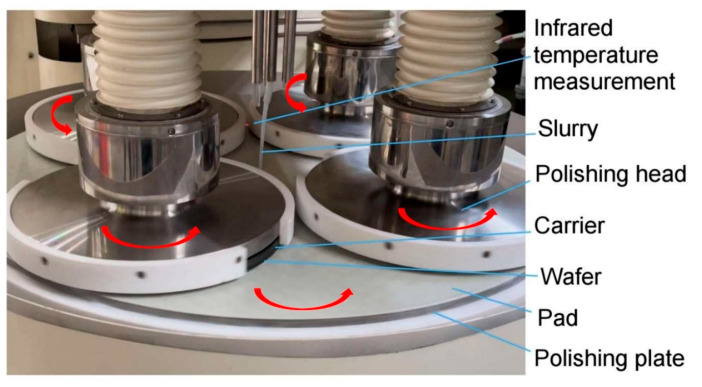
Single-sided chemical mechanical polishing diagram of a silicon wafer.

As shown in Figure 8, in the processing area of chemical mechanical polishing of four polishing head silicon wafers, one side of the polishing plate is attached to a polyurethane polishing pad, and the silicon wafer is attached to the carrier as a processing workpiece with a wax coating, that is, on a high-precision SiC ceramic substrate. The ceramic carrier is pressed under the polishing head, and the four polishing heads are in a symmetrical uniform state. The number of silicon wafers attached to a ceramic carrier under each polishing head varies according to the size and quantity of silicon wafers. The 5-inch silicon wafers tested in this paper are attached with 5 wafers of the same thickness to each ceramic carrier, and a total of 20 wafers are processed under full load in a batch. The CMP polishing liquid was a SiO_2_ abrasive suspension with KOH alkaline solution added. Both the polishing plate and the polishing head are rotated in the same direction at approximately 60 rpm. The polishing slurry flows down the middle, flows into the processing area under the guidance of the rotation of the polishing plate and the polishing head and the groove of the polishing pad, and then returns to the polishing liquid barrel. As shown by the red arrow in Figure 8, the polishing plate and polishing head rotate in the same direction. An infrared thermometer is installed on the polishing pad to monitor the temperature on the polishing pad in real time [31,32,33]. An infrared thermometer was used to detect the polishing slurry on the pad during processing. In the experiment, constant temperature control of the polishing slurry was used, which can maintain a constant temperature of the polishing slurry in multiple experiments. This avoids the influence of the temperature increase caused by processing heat on the uniformity of removal in multiple consecutive experiments.

**Figure 9:**
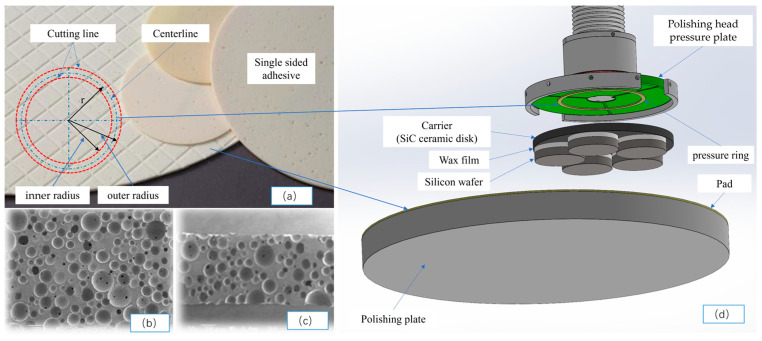
Schematic diagram of pressure ring production and bonding. (**a**) Polishing pad and pressure ring production. (**b**) ×50 SEM image of pad surface. (**c**) ×50 SEM image of pad cross section. (**d**) Bonding of pad and pressure ring.

Figure 9 shows a schematic diagram of the production of the pressure ring, which is made of MH™ series polishing pads, NITTA DuPont INCORPORATED [34]. As shown in (b) and (c), it is an elastic material with loose pores. The material of the polishing pad is mainly polyurethane, with a thickness of 1.3 mm, a Shore hardness of A84, and a density of 0.52 g/cm^3^. It is cut according to the required inner and outer diameters and then bonded to the polishing head pressure plate via the single-sided bonding function of the polishing pad.

The main dimensions related to the experiment include two sizes of pressure rings: (a) an outer radius of 110 with an inner radius of 100 mm and (b) an outer radius of 120 with an inner radius of 110 mm. The carrier radius is 175 mm, the wafer radius is 62.5 mm, and the pad radius is 457 mm.

If the polishing results of a group of silicon wafers under a certain polishing head differ in thickness near the center and edge of the carrier, the usual approach is to experiment with several pressure rings, compare the results, and adopt a better method. Owing to the establishment of pressure ring adjustment Formula (19), when the edge thickness t_p4_ is greater than the thickness t_p2_ at the center of the carrier, the pressure ring will need to be enlarged; conversely, it will need to be reduced. The formula can calculate the direction and size of the adjustment, reducing the number of comparative experiments. The following is an example of verifying the adjustment of a polishing head pressure ring, which was verified through simulation and experiment, and the exponent of the adjustment formula was obtained.

**Figure 10:**
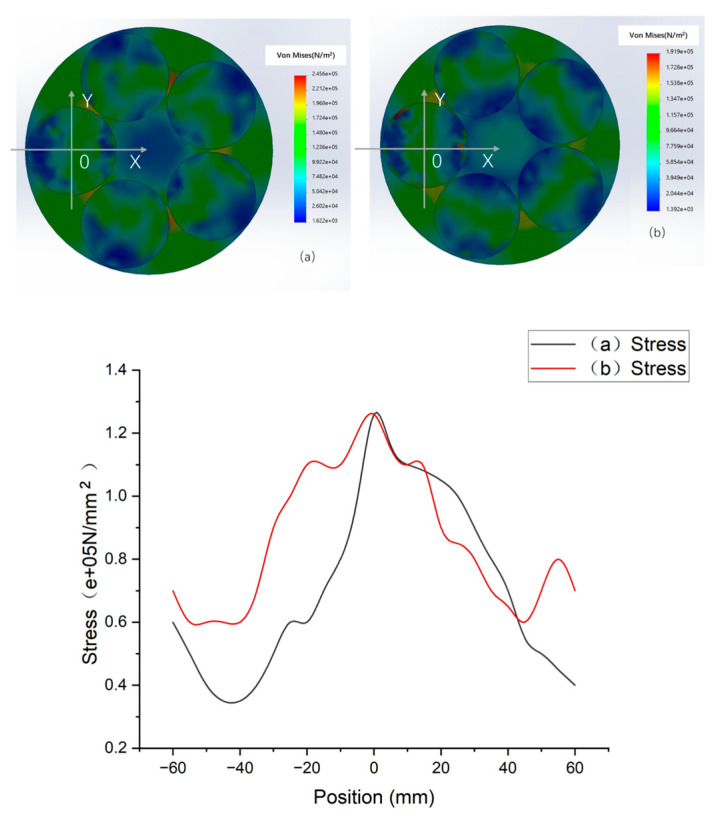
Stress contrast diagram of two pressure rings.

Figure 10 compares the stress distributions under two different pressure rings under the condition of 1000 N applied to each polishing head. (a) is a pressure ring with an inner radius of 100, and the influence of the pressure ring is closer to the center of the carrier and the inside of the silicon wafer. (b) is a pressure ring with an inner radius of 110, and the effect of the pressure ring is more centered homogenization on the silicon wafer; that is, the stress uniformity (b) is better than that in (a).

The following is a comparison of the silicon wafer thickness and TTV before and after the polishing experiments were conducted to improve the common results of the two factors after optimizing the process of silicon wafer bonding and adjusting the pressure ring. Among them, W1~W5 are pre-improved, whereas W6~W10 are optimized silicon wafers.

According to Formula (15), the TTV can be calculated with one line of data for each silicon wafer.

$$\begin{array}{c} {TTV}_{1}=Max\left(t_{11},t_{12},t_{13},t_{14},t_{15}\right)-Min\left(t_{11},t_{12},t_{13},t_{14},t_{15}\right)\\ =532.28-529.58=2.70\;\mu m. \end{array}$$

According to this method, TTV2~TTV5 can be calculated as 2.14, 2.53, 2.54, and 2.57, respectively.

$\bar{TTV}$ is calculated according to Equation (16):

$$\begin{array}{c} \bar{TTV}=({TTV}_{1}+{TTV}_{2}+\cdots +{TTV}_{n})/n\\ =\frac{2.70+2.14+2.53+2.54+2.57}{5}=2.496\;\mu m \end{array}$$

σ is calculated according to Equation (17):

$$\sigma =\sqrt{\frac{{{(TTV}_{1}-\bar{TTV})}^{2}+{{(TTV}_{2}-\bar{TTV})}^{2}+\cdots +{{(TTV}_{n}-\bar{TTV})}^{2}}{n-1}}$$

$$\begin{array}{c} =\sqrt{\frac{{\left(2.70-2.496\right)}^{2}+{\left(2.14-2.496\right)}^{2}+{\left(2.53-2.496\right)}^{2}+{\left(2.54-2.496\right)}^{2}+{\left(2.57-2.496\right)}^{2}}{5-1}}\\ =0.21\;\mu m \end{array}$$

If σ meets the requirements of CPK, we can determine the relationship between the same point data by determining the average value of the column data.

The AVETHK data in Table 2 are consistent with situation (1); that is, the values of $\bar{t_{p1}}$, $\bar{t_{p3}}$, and $\bar{t_{p5}}$ are close, and $\bar{t_{p4}}<\bar{t_{p1}}<\bar{t_{p2}}$.

$$\bar{t_{p2}}-\bar{t_{p4}}=532.04-529.54=2.5\;\mu m$$

**Figure 11:**
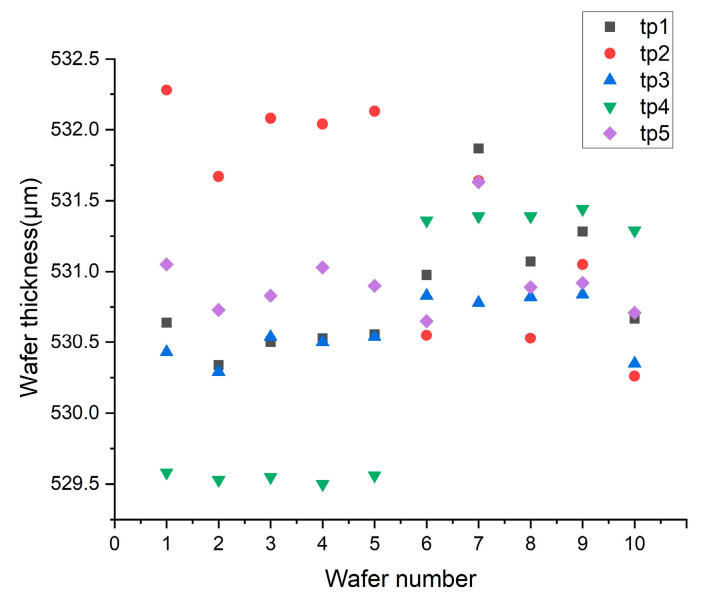
Wafer thickness comparison diagram.

Through experiments, the outer radius of the pressure ring is increased to 120 mm, and the inner radius is increased to 110 mm. For comparison, the thicknesses of the improved silicon wafer numbered 6~10 and the previous silicon wafer thicknesses numbered 1~5 are shown in Figure 11.

Numbers one to five in Figure 11 are the thickness values before pressure ring adjustment. The tp4 data shown in green are smaller than the tp2 data shown in red, whereas the tp1, tp3, and tp5 data are close, indicating that the removal rate of the silicon wafer at point 4 close to the center of the carrier is higher than that at point 2 due to greater stress. By increasing the diameter of the pressure ring, the thickness values obtained from the silicon wafers numbered 6 to 10 become more concentrated. This figure shows that the stress distribution can be improved by adjusting the pressure ring, thus improving the removal uniformity.

**Figure 12:**
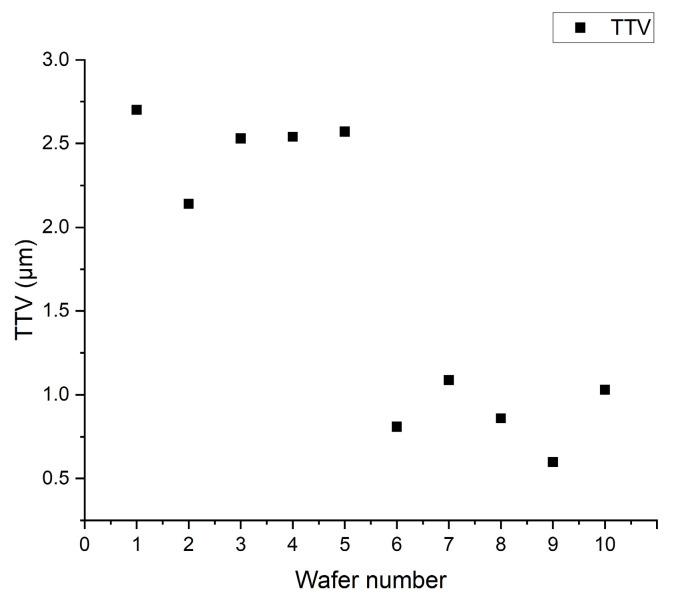
Wafer TTV comparison diagram.

Figure 12 shows the TTV comparison diagram of the silicon wafers. Numbers one to five on the left side of the figure are the TTVs of the silicon wafers before pressure ring adjustment, which are between 2.0 and 3.0 μm. Numbers 6 to 10 on the right are the adjusted values of the pressure rings, which range from 0.5 to 1.25 μm. This figure shows that the TTV can be significantly improved by increasing the stress distribution uniformity by adjusting the pressure ring.

Therefore, we can substitute the adjusted pressure ring data into Equation (19):

$$\frac{115-105}{100}={\left(\frac{2.5}{529.52}\right)}^{\lambda }$$

Solve the above equation, and we obtain λ = 0.43.

If the pressure ring needs to be adjusted in the future, it can be calculated by the measured thickness value. This method can be used as a reference method for pressure ring adjustment and reduce the number of pressure ring comparison tests.

On the basis of the experimental results, the discussion is as follows:

(1) The pressure ring is the optimization result of adjusting the polishing head in this experiment. However, owing to the slight differences in the flatness of the polishing head and the pressure plate of each polishing head, personalized adjustments can be made in this way when the pressure distribution of individual polishing heads is uneven. The index λ of adjustment for each polishing head is different. Here, a method of adjustment is provided.
(2) The pressure ring is made of a polyurethane material with single-sided bonding ability, which is consistent with the polishing pad, has elasticity, and meets the clean standards required for semiconductor silicon wafer processing, making it easy to manage production. Experiments have shown that good removal uniformity adjustment effects have been achieved, but currently no other materials have been selected as pressure rings for comparative experiments.
(3) This pressure ring size adjustment formula can accommodate adjustments of 5 inches, as well as 4 inches and 6 inches. Owing to the different bonding positions of silicon wafers of different specifications, there may be differences in the index λ, and the principle and method of obtaining the index are the same. Notably, this multipiece polishing machine is commonly used for processing 4–6-inch silicon wafers. In principle, it can also be used for other wafers with a larger range of commercial sizes from 0.5 to 8 inches, whereas larger 12-inch silicon wafers are more commonly controlled by regional pressure distribution because of the use of centered single-piece polishing.
(4) This pressure ring adjustment method is used in situations where nonuniformity is removed from some silicon wafers with polishing heads. Current experiments have shown that the adjustment limit is when the uniformity thickness difference in the silicon wafer is within 1 μm.

## 4. Conclusions

The improvement in wax coating adhesion and pressure ring adjustment comprises two aspects that improve removal uniformity. First, the improvement in wax coating adhesion can increase uniformity by 20% under the same conditions of polishing equipment. This section omits separate experimental data and presents only the results. Further adjustment through the pressure ring can improve the uniformity of the removal of individual polishing heads. The final experimental result is the result of the joint improvement of two factors.

Firstly, a 3D model of a single-sided chemical mechanical polishing machine for processing small silicon wafers is established, and the deflection of the silicon wafers is calculated via a small deflection sheet model. For 5-inch silicon wafers, a deflection of 0.34 μm ≤ w ≤ 1.36 μm is generated. Through good boundary conditions, such as improving the bonding quality of silicon wafers, the ideal conditions for fixation can be approached to reduce deflection. By comparing the thickness of the wax film and the variance uniformity, a better recipe when the speed of the wax-coated coating is 1500 rpm, 2500 rpm, and 3000 rpm for 1, 2, and 3 s, respectively, can be obtained; that is, better bonding boundary conditions can be obtained, which is beneficial for improving the removal uniformity. Through comparative experiments, under the same equipment conditions, improving the bonding conditions can improve the TTV level by 20%.

Then, using different pressure ring sizes through compressive stress simulation can improve the removal uniformity. On this basis, comparative tests were conducted on pressure rings made of the same material as the polishing pad with different sizes. A comparison of the detection data of polished silicon wafers revealed that good removal uniformity results were achieved. An exponential calculation was performed on the size adjustment formula of the created pressure ring. This formula calculation method can obtain the optimal adjustment size target value for this polishing head in two experiments. This method can reduce the number of simple comparative experiments using multiple pressure rings, providing a reference method for adjusting the size of pressure rings.

This study not only changes the uniformity of stress distribution by adjusting the size of the pressure ring but also reduces the deformation of silicon wafers by improving the boundary conditions of small deflection deformation by changing the bonding uniformity. To achieve the purpose of jointly improving the removal uniformity, the TTV of 5-inch silicon wafer multichip polishing can be increased to less than 3 μm. The reliability of the repeatability is also verified through batch experiments, so it has reference significance for engineering applications involving the chemical mechanical polishing of silicon wafers to improve the removal uniformity caused by pressure.

## Figures and Tables

**Table 1:** Deflection calculation parameters.

Part Name	Material	Young’s Modulus/MPa	Poisson’s Ratio
Polishing head pressure plate	SUS316	200,000	0.394
Pressure ring	PU nonwoven fabrics	20	0.3
Carrier	SiC	400,590	0.22
Wafer	Silicon	190,000	0.278
Pad	PU nonwoven fabrics	20	0.3
Polishing plate	Invar	140,000	0.3

**Table 2:** Silicon wafer thickness test data (unit: μm).

	*tp*_11_	*tp*_12_	*tp*_13_	*tp*_14_	*tp*_15_
W1	530.64	532.28	530.43	529.58	531.05
W2	530.34	531.67	530.29	529.53	530.73
W3	530.50	532.08	530.54	529.55	530.83
W4	530.53	532.04	530.50	529.50	531.03
W5	530.56	532.13	530.54	529.56	530.90
AVETHK of W1~W5	530.51	532.04	530.46	529.54	530.91
W6	530.98	530.55	530.83	531.36	530.65
W7	531.89	531.64	530.78	531.39	531.63
W8	531.07	530.53	530.82	531.39	530.89
W9	531.28	531.05	530.84	531.44	530.92
W10	530.67	530.26	530.35	531.29	530.71
AVETHK of W6~W10	531.18	530.81	530.72	531.37	530.96

## Data Availability

The original contributions presented in this study are included in the article. Further inquiries can be directed to the corresponding author.
